# Generation of Gene Ontology benchmark datasets with various types of positive signal

**DOI:** 10.1186/1471-2105-10-319

**Published:** 2009-10-07

**Authors:** Petri Törönen, Petri Pehkonen, Liisa Holm

**Affiliations:** 1 The Holm Group, Biocenter II, Institute of Biotechnology, PO Box 56, 00014 University of Helsinki, Finland; 2 Department of Biological and Environmental Sciences, P.O. Box 56, 00014 University of Helsinki, Finland; 3 Department of Biosciences, P.O. Box 1627, 70211 University of Kuopio, Finland

## Abstract

**Background:**

The analysis of over-represented functional classes in a list of genes is one of the most essential bioinformatics research topics. Typical examples of such lists are the differentially expressed genes from transcriptional analysis which need to be linked to functional information represented in the Gene Ontology (GO). Despite the importance of this procedure, there is a little work on consistent evaluation of various GO analysis methods. Especially, there is no literature on creating benchmark datasets for GO analysis tools.

**Results:**

We propose a methodology for the evaluation of GO analysis tools, which consists of creating gene lists with a selected signal level and a selected number of independent over-represented classes. The methodology starts with a real life GO data matrix, and therefore the generated datasets have similar features to real positive datasets. The user can select the signal level for over-representation, the number of independent positive classes in the dataset, and the size of the final gene list. We present the use of the effective number and various normalizations while embedding the signal to a selected class or classes and the use of binary correlation to ensure that the selected signal classes are independent with each other. The usefulness of generated datasets is demonstrated by comparing different GO class ranking and GO clustering methods.

**Conclusion:**

The presented methods aid the development and evaluation of GO analysis methods as they enable thorough testing with different signal types and different signal levels. As an example, our comparisons reveal clear differences between compared GO clustering and GO de-correlation methods. The implementation is coded in Matlab and is freely available at the dedicated website .

## Background

The increasing usage of high-throughput methods is changing the biosciences. The analysis of the resulting data often generates a list of genes that fulfil certain selection criteria. Such a list can be, for example, a cluster of co-expressed genes, genes up-regulated in disease samples or genes representing a similar phenotype in a knock-out experiment. The resulting gene lists are often too large for direct manual analysis, and they regularly contain many false positive genes. Therefore, it is standard practice to use large scale functional classifications, like Gene Ontology (GO [1]) or MIPS funcat [2], to aid the analysis. These functional classifications in combination with statistical analysis methods enable the filtering of occasional false-positive genes, emphasize over-represented functional classes and generally facilitate the analysis (see, for example, [3-7]). This has taken the analysis of biological function from the single gene level to the more informative biological process level. Functional classes can also be used to select the optimal set of clusters [5], to find heterogeneity in the expression of functional groups [8], to evaluate clustering results [9], to analyze differential gene expression at the gene class levels [10], as an input data for the prediction of interacting genes [11], or to analyze the functional heterogeneity of the genes in the reported gene list [12]. Indeed, the field has seen an explosion of methods, which aim to report the most important functional features for a group of genes or proteins [7].

Despite its significant benefits, the standard enrichment analysis of functional classes has still some notable unsolved problems. Good quality annotation is one of the most critical requirements for class data analysis. This quality can be weakened by the **lack of information or disinformation in the class annotations **for the analyzed organism [7]. The functional class analysis also requires gene ID mapping. This is often a nontrivial task as there are frequently **ID matching and conversion problems **(referred also as Name-Space mapping between different databases [7]). The ID matching and conversion problems again increase the disinformation in the used annotations. One further challenge in the analysis of the over-represented functional classes is the existence of multiple biologically very similar GO classes. These are caused by **the strong correlations between the analyzed functional classes**. The repetitive occurrence of similar classes related to the same biological theme often masks other weaker but biologically equally relevant themes from the end user [12,13]. Further problems in the analysis with functional classes include the selection of a suitable null hypothesis [14], sharp binarization of continuous data for over-representation analysis [6] and others [15]. However, these are considered to be outside the scope of this manuscript.

The wealth of available GO analysis methods and the analysis related problems raise the need for detailed evaluation of the available GO analysis tools. Indeed, publications simply often report findings from some real-life datasets, by the promoted method. However, this does not unambiguously quantify the performance of the compared methods. Such quantification would require that we would clearly define positive and negative features and test methods on how well they can be separated.

There are two potential ways for a detailed evaluation of GO analysis. One can do the analysis using various very well known datasets, and test whether the obtained results correspond with earlier knowledge. The drawback of this method is that currently there are, to our knowledge, no datasets available, where all the reported functional classes could be classified clearly as either true or false positive. This makes it hard to quantify the performance of compared methods with real data. Furthermore, this method does not allow repetitive testing with a large number of positive and negative datasets. Another option would be to generate a large set of artificial datasets with both positive and random signal and to see how well the analysed method separates them. Although the generation of negative signal can be a simple random sampling from the gene pool, the generation of a positive test signal has not been clearly discussed in the literature.

In order to aid the comparison of various methods, the current work proposes a novel methodology for creating benchmark GO datasets with known over-represented (i.e. signal) classes. POSGODA (POSitive artificial GO DAta generator) takes as an input a large set of genes with GO classifications. This set can be, for example, all known genes in the analyzed genome or all the genes from a popular gene expression chip. Datasets can be created with varying size of gene lists, with varying level of over-representation signal and with varying number of independent signal classes. To compensate the larger overall signal, which automatically arises from the larger number of independent signal classes, POSGODA allows the choice between a number of methods for normalizing the overall signal. Note that by starting with a real classification, the generated datasets will represent all the features present in the real data, such as class correlation and hierarchical class structure. An earlier work [16] used the term *plasmode dataset *to separate similar gene expression datasets from purely artificial datasets.

The inputs for POSGODA are the signal range of p-values, the size of the output gene list, and the number of independent signal classes. Its outputs include a list of independent GO terms, selected as positive, a gene list which shows signals in the selected classes, and the p-value signal embedded in the GO class. POSGODA takes the input signal range of p-values and, if required, adjusts them using the number of signal classes. Next, it selects the independent signal classes from the randomly ordered GO class list. These independent classes are tested, one at the time, looking for the class over-representation level that is closest to the selected signal level. If the resulting signal from the class deviates too much from the desired signal level, the search is redone a few times with the same class, before moving to the next class in the class list. This is repeated until the desired number of signal classes is reached. Once the signal classes have been defined, POSGODA creates the gene list, with the required number of members from each signal class. If this resulting gene list is smaller than the desired gene list size, POSGODA adds randomly selected false positive genes to obtain the required size.

Related work has been presented before [13]. However, there are major differences. *i*) POSGODA defines the added number of class members based on the resulting hypergeometric p-value, which has to be within the signal range given by the user. Earlier works defined this as raw percentage of class size, which will generate different signal levels for different size of classes and gene list. *ii*) POSGODA controls the potential correlations between the classes that yield a positive signal and furthermore the potential correlation between GO terms via a third intermediate class. These limitations ensure that we generate two separate signals instead of multiplying the same signal twice, and thus generating a single stronger signal. *iii*) POSGODA includes various methods that can be used to scale the signal, when embedding it to a larger number of signal classes. Here, the scaling helps the positive signals to stand out from the large pool of random signals. However, it should noted that POSGODA can be used also without the signal scaling.

We hope that POSGODA will improve GO analysis especially by allowing the repetitive testing of tools on artificial datasets. These enable the evaluation using statistical methods, like the Receiver-Operating Characteristics (ROC) curve or T-test to quantify the separation between positive and negative features or datasets. Furthermore, the analysis can be repeated with different signal parameters, to see areas of optimal performance for various methods. In addition, we highlight specific topics in which POSGODA can be used:

• The generated datasets can be used to test the existing or novel methods that filter correlations from GO structure [12,13,17]. This shows how well they are able to report positive GO classes or GO classes that are strongly linked to positive GO classes.

• The generated datasets can be used to test GO data clustering methods [12,18], as genes belonging to different positive classes are expected to represent separate clusters or cores for clusters. Datasets can be used also to test clustering of GO classes.

• Evaluating how well asymptotic methods (Chi square, log likelihood etc.) approximate the hypergeometric test.

• Testing and evaluating methods and their parameters during method development.

It is also relevant to notice that there are two issues that POSGODA cannot evaluate. These are the correctness of the input GO data matrix, and the testing of enrichment scoring functions that are potentially better than the hypergeometric p-value.

We demonstrate the usefulness of POSGODA by generating a large pool of datasets with positive signals for testing various GO analysis tools. The main emphasis in our comparison is on the evaluation of various methods that filter correlation from the GO structure. Our results show significant variation between methods in their ability to emphasise the independent over-represented GO classes.

## Results

Due to the nature of the current work, the results section first shows some of the features of the positive datasets generated by the methodology. We briefly describe the workflow of POSGODA, as a part of the results. The details on each step are given in the Materials and Methods. Further details are given in supplementary text. We also demonstrate the usage of the generated datasets in two comparisons. The methods, some of the results from the performed comparisons and demo datasets are available from our web site .

### Complete method workflow

POSGODA requires from the user a binary GO matrix (genes in rows, classes in columns), where one denotes the membership of the gene in the specific class. With this real GO dataset we make sure that our data has the type of structure that actual biological datasets also have. POSGODA also requires the user to calculate the *N*_class _* *N*_*class *_correlation matrix that represents the correlation of the GO classes, and an estimate for *effective number*, *N*_*eff *_[19,20]. *N*_*class *_refers here to the exact number of classes, whereas *N*_*eff *_refers to the estimated actual number of independent classes (see materials and methods for details). The correlation matrix is used to evaluate the independence of the signal classes and *N*_*eff *_is used to scale the signal levels. A standard matrix rank function is used for the estimation of *N*_*eff *_throughout the current work.

The user is required to input the minimum and maximum p-values, which define the signal range; the size of the reported gene list; the number of signal classes; and the method for normalizing the signal with multiple classes. Here normalization refers to inverse function for selected multiple testing correction.

**Figure 1:**
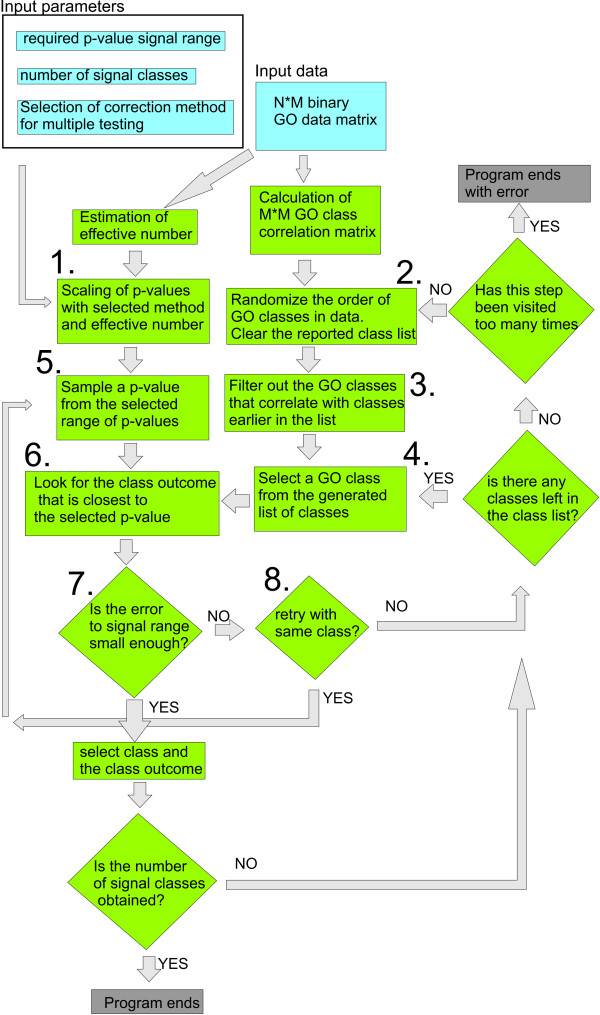
**Flow chart representation of the first steps of POSGODA**. A simplified representation of the flow chart of the function used to select independent classes, to define the multiple testing corrected p-value signal levels, and to define the class outcome closest to the signal level. Input parameters and data are represented with light blue, intermediate steps are represented with green and end states are represented with gray. Steps associated with numbers are explained in the text. For more detailed un-simplified representation see the supplementary text.

The first steps of POSGODA are shown in figure 1. A more detailed description in pseudocode is given in the supplementary text S1 [see additional file 1]. POSGODA starts by normalizing the minimum and maximum p-values, given by the user (step 1. in figure 1.). The normalization depend on the effective number and the selected signal normalization method. Then POSGODA selects suitable functional classes by looking at the randomly ordered classes, and excluding classes that have unwanted correlation with classes already included in the class list (steps 2. and 3. in figure 1.). The resulting GO class list will be then used as an input to step 4. A target signal level is randomly selected between the minimum and maximum p-values (step 5. in figure 1.) After this, POSGODA tests whether it is possible to obtain a close enough signal with the selected class (steps 6. and 7.). This might not be possible with classes having very few members or classes that include practically all the genes. If the search fails, new signals, sampled from the signal range, are tested with the same class a few times (the yes return from the step 8. to step 5.), before moving to the next class in the class list (the return to step 4.). This algorithm reports the required number of genes from one class for the suitable signal level. If the desired number of signal classes cannot be found, the class list is re-sampled and the whole search process is started again (the return to step 2.). As different classes have a different number of correlating classes, the order in which the classes are selected in the random process affects the number of available independent GO classes. However, in most of our tests, the first or second random list of classes generated adequate results.

**Figure 2:**
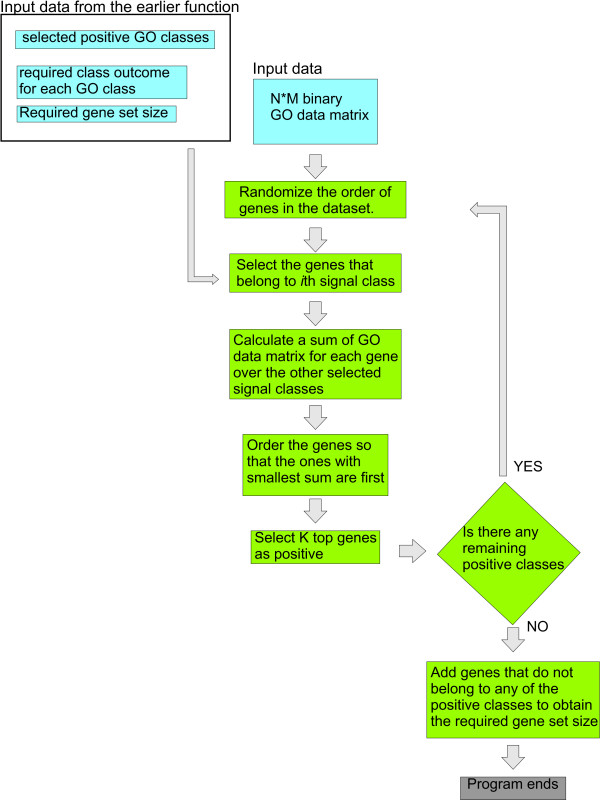
**Flow chart representation of the last steps of POSGODA**. A simplified representation of the flow chart of the function used to map genes to the generated gene list. Colouring is similar to fig. 1. The detailed representation is in the supplementary text.

The final steps of the workflow are shown in figure 2. These steps select the actual genes to the generated gene list. Here, the required number of genes is selected from the class members to the gene list. Selection favours genes that are not members of any other signal class. This avoids the addition of extra members to other signal classes, while selecting members for one class. Finally, the size of the resulting gene list has to be controlled. Too small a gene list is corrected by adding genes that do not belong to any of the selected signal classes (false positive genes) to the gene list. Too large a gene list is corrected by redoing the whole search process.

As an output, the method reports the binary vector for genes represented by the GO matrix, having one for the genes that were selected to the gene list. Also a list of column numbers for the classes having the positive signal is reported. Furthermore, our supplementary functions can be used to print the corresponding gene names or positive class names to text files. The whole program is coded in Matlab (Mathworks inc.). The purpose of using a script language is that other method developers could easily create scripts for repetitive testing of various methods and also to allow easy modification of the generated method.

### Signal level evaluation

Throughout the first analysis steps we use GO data for a subset of the yeast genome (regulated genes selected in [5]) with all the three subsets of GO (July 2005). We use a gene list (cluster) size of 300 genes and matrix rank to estimate *N*_*eff*_. We also calculated the binary correlations for this test dataset. The dataset, the calculated correlations and *N*_*eff *_estimates are available in the supplementary web page. Note that the results should not be affected by the selection of different species, selecting various subsets of the genome pool or using only one subset of GO or even a totally different hierarchical classification dataset.

First, we wanted to know if we are able to create the correct signal level for the selected signal classes, when we scale the signal using the selected p-value combination methods. To confirm this, we produced 200 datasets with *i*) 5 or 10 signal classes *ii*) using FDR or Holm's method for signal normalization *iii*) with p-value signal ranging from 0.01 to 0.05. The process creates altogether 2000 signal classes for evaluation, when 10 signal classes was used and 1000 classes when 5 signal classes was used. Table 1 summarizes the results for each dataset. What we hope to see is that when we reverse the signal normalization, we would have p-values in the aimed signal range. Table 1 shows the selected percentiles from each dataset for p-values calculated for signal GO classes with and without the signal normalization.

**Table 1:** Evaluation of embedded signal levels with two signal normalizations

			**selected percentiles**						
**M.T. meth**.	**Signal** ** Classes**		**0**	**5**	**25**	**50**	**75**	**95**	**100**
Holm	5	uncorr.	2.54E-05	2.01E-05	1.59E-05	9.99E-06	5.8E-06	4.73E-06	4.26E-06
Holm	10	corrected	0.0556	0.044	0.0348	0.0219	0.0127	0.0104	0.0093
Holm	5	uncorr.	2.54E-05	2E-05	1.58E-05	9.99E-06	5.58E-06	4.73E-06	4.18E-06
Holm	10	corrected	0.0555	0.0437	0.0346	0.0218	0.0122	0.0103	0.0091
FDR	5	uncorr.	0.000124	0.000107	8.69E-05	5.38E-05	4.04E-05	2.75E-05	0.000021
FDR	10	corrected	0.054	0.0481	0.044	0.0281	0.0191	0.0114	0.0093
FDR	5	uncorr.	0.000246	0.000219	0.0002	0.000128	8.69E-05	0.000052	4.23E-05
FDR	10	corrected	0.0547	0.0468	0.0382	0.0236	0.0177	0.0121	0.0092

Table shows the summary of results with reported percentiles. Signal generation is tested with 5 and 10 positive classes and with signal normalizations that use Holm's method and FDR. Note that 5 percentile limit and 95 percentile limit stay within the required 0.01-0.05 range. Minimum and maximum show deviation from the signal range but the deviation is always only appr. 10% of the allowed signal.

The results show that 90% of the data fall within the aimed 0.01 - 0.05 signal range. Furthermore, the deviations from this signal range are quite small. The deviations are approximately 10% from the aimed signal level, which should not disturb the analysis based on these datasets. Note that we are bound to have some error as we are trying to match a discrete variable (the number of class members in a cluster) with a continuous signal level. Therefore POSGODA has been designed to allow a small error from signal range.

### Signals obtained from one example dataset

The signal that is obtained from the data is illustrated in detail with the top-scoring GO classes for one of the datasets in table 2. Data was produced with FDR normalized signal levels and with five signal classes. The required signal level was set to [0.01-0.05]. Table 2 shows the reported classes. What can be seen immediately is that embedding a signal in one class usually causes a similar signal also in other similar classes. These classes show strong correlation with the original signal class. The repetitive occurence of GO classes representing very similar functions is often also noticed in the real datasets [12]. These results confirm the benefits of filtering correlating classes, as many of the strongly correlating classes represent practically the same signal. Without the filtering, one could have selected repetitively these almost identical classes as signal classes, resulting in the multiplication of the same signal. Furthermore, one can see that the number of classes with strong correlations varies significantly. For example, we see four classes having correlation stronger than 0.9, and additionally three classes having correlation stronger than 0.8 with class 'RNA ligase activity', whereas the class 'response to inorganic substance' has not got any correlations with other classes in the result list. Table 2 also demonstrates that the selected signal classes do not correlate with each other. This is natural as POSGODA does not accept signal classes that are correlated with each other.

**Table 2:** Top scoring GO classes from one of the positive datasets

			**Correlation with signal classes**				
**cl. Num**.	**log10(p)**	**Class names**	**13**	**12**	**9**	**5**	**6**
1	5.152	metal ion transporter activity	-0.005	-0.0078	-0.0068	-0.0067	**0.9068**
2	4.8916	carboxylic acid transport	0.0266	-0.0088	-0.0076	**0.8561**	0.0165
3	4.7933	organic acid transport	0.0262	-0.0089	-0.0077	**0.8475**	0.0162
4	4.6381	tRNA ligase activity	-0.0048	-0.0075	**0.9863**	-0.0064	-0.0064
5	4.6381	**amino acid transport**	-0.0048	-0.0075	-0.0065	**1**	-0.0064
6	4.6381	**di-, tri-valent inorganic cation transporter activity**	-0.0048	-0.0075	-0.0065	-0.0064	**1**
7	4.6381	ligase activity, forming carbon-oxygen bonds	-0.0048	-0.0075	**0.9863**	-0.0064	-0.0064
8	4.6381	ligase activity, formingaminoacyl-tRNA and relatedcompounds	-0.0048	-0.0075	**0.9863**	-0.0064	-0.0064
9	4.5219	**RNA ligase activity**	-0.0048	-0.0076	**1**	-0.0065	-0.0065
10	4.2093	carboxylic acid transporter activity	0.0269	-0.0087	-0.0075	**0.7439**	0.0168
11	4.1976	ligase activity, forming phosphoric ester bonds	-0.005	-0.0079	**0.9615**	-0.0068	-0.0068
12	4.1196	**bud tip**	-0.0056	**1**	-0.0076	-0.0075	-0.0075
13	4.0201	**response to inorganic substance**	**1**	-0.0056	-0.0048	-0.0048	-0.0048
14	3.9477	organic acid transporter activity	0.0258	-0.0089	-0.0078	**0.7214**	0.0159
15	3.8469	transition metal ion transporter activity	-0.0042	-0.0066	-0.0057	-0.0057	**0.8813**
16	3.7791	iron ion transporter activity	-0.0025	-0.0039	-0.0034	-0.0034	**0.5258**
17	3.6126	tRNA aminoacylation for protein translation	-0.0044	-0.0068	**0.8395**	-0.0059	-0.0059
18	3.6126	amino acid activation	-0.0044	-0.0068	**0.8395**	-0.0059	-0.0059
19	3.6126	tRNA aminoacylation	-0.0044	-0.0068	**0.8395**	-0.0059	-0.0059
20	3.5079	ion transporter activity	-0.0092	-0.0144	-0.0125	-0.0123	**0.5218**
21	3.4751	amine transport	-0.0055	0.0121	-0.0075	**0.8651**	-0.0074
22	3.3985	amino acid transporter activity	-0.0045	-0.0071	-0.0061	**0.9128**	-0.0061
23	3.2561	cation transporter activity	-0.0085	-0.0134	-0.0116	-0.0115	**0.5605**
24	3.246	di-, tri-valent inorganic cation transport	0.0258	-0.0089	-0.0078	0.0159	**0.7685**
25	3.1866	transporter activity	-0.0055	-0.0192	-0.023	0.2493	0.2833
26	3.1644	transition metal ion transport	0.0291	-0.0082	-0.0071	0.0186	**0.6841**
27	3.0334	basic amino acid transporter activity	-0.0022	-0.0035	-0.0031	**0.4702**	-0.003
28	2.9412	siderophore-iron transporter activity	-0.0016	-0.0025	-0.0022	-0.0021	**0.3324**
29	2.9412	siderophore transporter activity	-0.0016	-0.0025	-0.0022	-0.0021	**0.3324**
30	2.9035	metal ion transport	0.0241	-0.0094	-0.0081	0.0144	**0.733**

Table shows 30 most over-represented GO classes from the dataset, ordered with the log10(p-values). Dataset was generated with artificial signal embedded to five classes, shown with bold font in the class list. Table shows also correlation of each reported class with these signal classes. The clear strongest correlation is shown here with bold font. Signal classes are marked in columns using their ranking in the result list. Notice the varying number of strong correlations that different signal classes have.

### Comparison of different GO class ranking methods

Here we demonstrate the usefulness of POSGODA by comparing different GO class ranking methods. We compare the ability of different methods to report positive classes among their top *k *classes (*k *= 1, 2..50). We compare three methods: Standard GO class list obtained from the DAVID server [21], and the parent-child [17] and the topology-elimination [13] algorithms implemented in Ontologizer software [17].

It is current standard to analyze the obtained results from class over-representation methods using the number of positive classes over different ranks in the sorted class list [13,17]. However, we take a slightly different approach and focus on the *difference in the cumulative sum of positive classes *between two compared methods. This analysis puts emphasis on the difference in results, rather than the actual results. Furthermore, we plot the various percentiles (0, 25, 50, 75, 100) in order to show how stable the difference is between the compared methods.

Most over-representation analysis methods may report a class that is close in the GO class structure to the positive class instead of the exact positive class. We considered that these should be also included in the evaluation. However, we wanted to down-weight their contribution to the final result. Therefore, we decided to weight these classes according to their correlation with the correct signal class. This is a unique, simple and intuitive method that automatically evaluates how similar the reported class is to the correct signal class.

**Figure 3:**
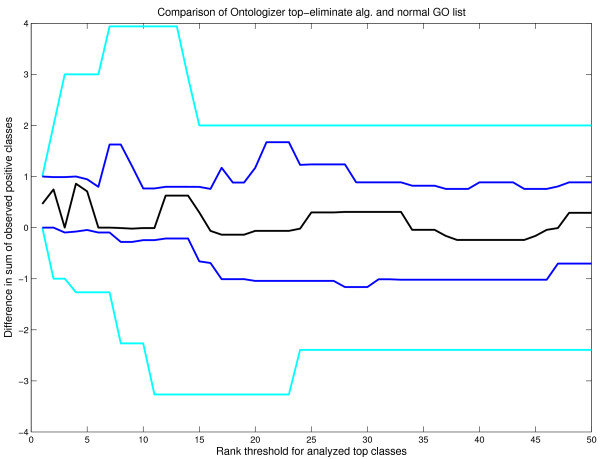
**Comparison of topology-elimination algorithm and standard ranked list**. A performance comparison between standard GO list from DAVID and Ontologizer output with the topology-elimination algorithm. We show the difference in the cumulative sum of positive GO classes across the top ranks. X-axis shows the rank, whereas the Y-axis shows the difference. Positive value indicate better performance by the Ontologizer algorithm and negative value better performance by the DAVID GO list. We summarize the comparison with 20 datasets showing five percentiles for each rank position: median (black line), 25 and 75 percentile (blue line) and minimum and maximum (cyan line). Notice that topology-elimination shows better performance across the top-15 ranks.

**Figure 4:**
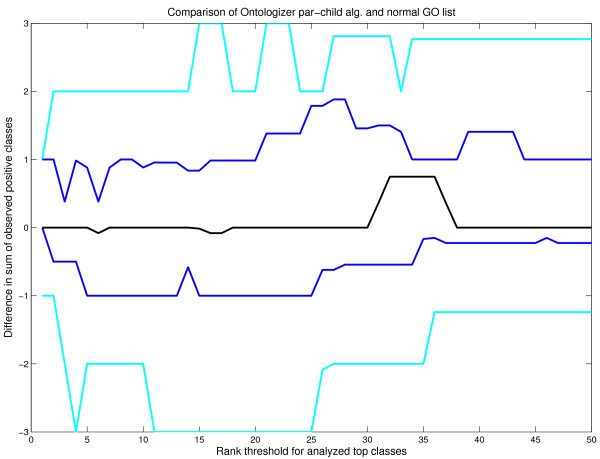
**Comparison of parent-child algorithm and standard ranked list**. A performance comparison between standard GO list from DAVID and Ontologizer output with parent-child algorithm. X and Y axis are similar to figure 3. Positive value refers to better performance by Ontologizer algorithm and negative value better performance by DAVID GO list. Percentiles and their colouring is also identical to earlier fig. 4. Here the difference, in favor of the parent-child algorithm seems small.

Figures 3, 4 show differences in cumulative sums, when both Ontologizer algorithms are compared with the standard GO list. Figure 3 shows that the topology-elimination algorithm clearly outperforms the standard list, especially across the top-15 ranks. This is highlighted by the 25^*th *^percentile, showing that in three quarters of the test runs topology-elimination outperformed the standard sorted GO list.

Figure 4 shows the corresponding results for the parent-child algorithm, a default algorithm in Ontologizer. Here the difference is surprisingly smaller than with topology elimination. Especially the median line shows zero difference between the two methods. This result seems to contradict earlier comparisons of these algorithms [17].

In summary, the comparison suggests that the best performance is obtained with the topology-elimination algorithm whereas the difference between parent-child algorithm and standard GO list was not clear cut. However, this analysis should be repeated using different parameters for artificial data generation to further confirm the results.

### Comparison of two GO data clustering methods

We further demonstrate the usefulness of POSGODA by demonstrating its use in the comparison of GO clustering methods. Here our aim is to see how well the generated clustering can separate the embedded signal classes, and also to monitor how large a portion the positive classes, or positive clusters, form of the clustering output.

We selected two algorithms, with different approaches, for our evaluation. One method, called GENERATOR [12], clusters genes using the available GO data. These gene clusters are then used to look for over-represented GO classes (see [12] for more details). Another method, implemented in the DAVID webserver [18], clusters the same data, but instead of clustering genes it clusters GO classes. It combines the correlating classes together as a single cluster. Both methods aim to represent the heterogeneity of the reported functional annotations and to limit the number of correlating classes that are seen with the over-represented classes.

There is no fixed way currently to the analysis GO clustering tools. Therefore, we propose a few simple rules for evaluation:

• GO clustering tools try to predict the positive classes. Therefore we have used prediction related measures, Positive Predictive Value (PPV), sensitivity and F1 score, to quantify the prediction performance.

• We selected three top GO classes from each cluster as the result, i.e. the predicted positive GO classes.

• Although we ranked clusters within the clustering, we decided to treat them here as equally good.

We omit details from our analysis. Briefly, GENERATOR generated between 6 to 13 clusters, whereas DAVID generated between 42 to 72 clusters. This suggests that GENERATOR clustering is closer to the correct cluster number, five. DAVID, on the other hand, usually ranked the positive class better in the ranked list of the reported clusters. We summarize the results in table 3. The table shows the portion of true positive GO classes among the predicted positive classes, represented by PPV (Positive Predictive Value), and the portion of predicted positive classes of the positive classes, represented by sensitivity. These two measures are combined in the F1 score. The results suggest that GENERATOR performs clearly better with this type of datasets. Although its sensitivity is slightly smaller, the PPV for GENERATOR is frequently ten times better. Weaker sensitivity might result from the selection of single clustering level from the GENERATOR output for the evaluation (see Materials and Methods for details). However, this could be corrected in manual analysis by monitoring several levels from the reported non-nested hierarchical clustering [12].

**Table 3:** Evaluation of DAVID and GENERATOR clustering using the generated test datasets

**dataset num**.	**PPV GEN.**	**PPV DAVID**	**Sens. GEN.**	**Sens. DAVID**	**F1 GEN.**	**F1 DAVID**	**GEN. -DAVID**
1	0.3	0.09	0.6	1	0.4	0.17	0.23
2	0.38	0.06	0.75	1	0.5	0.12	0.38
3	0.38	0.07	0.6	0.8	0.46	0.13	0.33
4	0.36	0.04	0.8	0.4	0.5	0.07	0.43
5	0.33	0.07	0.6	1	0.43	0.14	0.29
6	0.23	0.05	1	1	0.38	0.1	0.28
7	0.14	0.04	0.33	0.67	0.2	0.07	0.13
8	0.15	0.02	0.67	0.33	0.25	0.04	0.21
9	0.36	0.03	0.8	0.4	0.5	0.05	0.45
10	0.38	0.09	0.6	1	0.46	0.16	0.3
11	0.67	0.07	0.8	0.8	0.73	0.13	0.59
12	0.42	0.05	1	0.6	0.59	0.09	0.5
13	0.43	0.07	0.6	0.8	0.5	0.13	0.38
14	0.5	0.04	1	0.5	0.67	0.07	0.6
15	0.5	0.09	1	1	0.67	0.16	0.51
16	0.33	0.03	0.67	0.67	0.44	0.06	0.38
17	0.09	0.03	0.33	0.67	0.14	0.07	0.08
18	0.42	0.07	1	1	0.59	0.13	0.46
19	0.38	0.05	0.75	0.75	0.5	0.09	0.41
20	0.13	0.03	0.33	0.67	0.18	0.06	0.12

Table presents the Positive Predictive Value (PPV), sensitivity (sens.) and F1 score for GENERATOR(GEN.) and DAVID clustering results. Furthermore, we show the difference of F1 scores between two methods. Notice that the F1 score for GENERATOR is consistently better, as is shown by the difference in the last column. DAVID outperforms GENERATOR only in sensitivity, but this is natural as DAVID created 3-10 times more clusters.

## Discussion

We have presented a methodology (POSGODA) with supplementary tools for generating positive GO datasets. The results suggest that POSGODA generates datasets with the required signal. We present the analysis of effective number, a variable used for scaling the signal, similarly to earlier work [20]. However, we have used here a simpler approach, implementing the matrix rank function. Currently, it still seems to be common to use the number of classes as the normalizing variable, although the number of classes ignores the correlations in the GO datasets, and is often bound to create a too strong correction to signal levels. Our results in table 2 illustrate this, as randomly selected classes can have up to 7 strong correlations.

POSGODA includes a simple unique evaluation of the independence of the GO signal classes. This evaluation is based on the correlation of the GO classes [5]. This measure is independent from the graph distance between the GO classes and simply answers to the question: How similar are the classes being compared? The measure can be used also to compare, say, MIPS functional classifications, SwissProt keywords and GO classifications with each other. Also, the whole proposed methodology could be similarly used with any binary classification matrix or combination of binary matrices.

There are a number of ways to use POSGODA. It could be used to evaluate methods on how close the reported p-values get to the p-value signals used in the POSGODA data generation. However, such analysis would require that the data matrix used by POSGODA comes from a very reliable source. The correctness of signal levels also depend on the *effective number *and the used signal correction method. Furthermore, potential differences could be caused by the exclusion of some GO evidence codes in the evaluated method and there are no 'golden rules' stating which evidence codes should be included in the analysis and which should be excluded. Therefore, we chose to monitor the rank of positive GO classes in the reported GO class list. Ranked list analysis is a robust approach that omits the actual p-value scores. It also resembles the explorative analysis frequently done with GO analysis tools. Our unique and simple addition to this analysis was the inclusion of GO classes that show strong correlation with one of the positive classes, as also positive. However, we weighted them using the correlation with the correct positive class. This is a novel, simple and intuitive way to downweight those classes as weaker hits than the correct positive classes.

Our comparison also included methods that generate clusters from GO data. Here we again looked for the ability of the methods to select the signal classes or other class that correlates strongly with a signal class. The results suggest, with clear difference, that the clustering created with GENERATOR is better than the clustering by DAVID. However, the results might change when testing with different dataset parameters and if false positives and false negatives are not weighted equally.

The compared tools used different versions of GO, with the DAVID server having GO structure from Jan 2008. The differences between this and newer GO structures might explain part of the differences between methods. However, out of all the classes that were in our test dataset, less than a half a percent was missing from the oldest GO structure (DAVID server). Furthermore, this effect is lessened in our analysis by counting also strongly correlating classes as positive. This helps, as the old version of GO structure has most likely already neighboring classes which correlate with added class.

POSGODA has potential further applications. One could link the genes with differential expression score and compare the various GO based threshold free gene expression analysis methods. This would simply require the selection of a potential model for positive and negative signal, like in some earlier research [22,23]. Our method differs from these two publications in that these works tested for the performance at the artificial GO class level, whereas our test datasets allows testing with the whole correlation structure of GO. We have implemented POSGODA for monitoring the separation between the positive and negative GO classes within each positive GO dataset. However, it would be interesting to test various methods that summarize the total signal across all the GO classes [8,9,24]. These methods could be tested by using positive datasets, generated by POSGODA, and similar random samples from the background population. Such analysis, however, would require testing of a large number of artificial datasets with varying parameters. Finally, we wish to point that the evaluation using artificial datasets cannot replace the evaluation done with real datasets, which should be used in combination with artificial datasets in evaluations.

## Conclusion

We hope that the proposed methodology would encourage the scientific community to more thoroughly test the various methods available for various functional genomics and GO datasets, and to stimulate discussion about the possibilities on testing these methods. Furthermore, we want to draw attention to some of the problems in the positive data generation, observed when using the real data GO matrix.

## Methods

### Problem setting

Our positive dataset generation is based on a few natural assumptions. The cluster of genes or gene list with positive signal is assumed to have true positive and false positive genes. True positive genes are assumed to represent biological functional group(s) (GO classes). The larger the portion of the positive genes, the stronger the associated signal. As a negative model, we consider a totally random sample from the total pool of genes, although this null hypothesis has been criticized [14]. We chose this random sampling for its simplicity. The other potential null and signal models should be easy to implement later in POSGODA. A single over-represented functional group from the hierarchical GO structure is expected to show the best match to our true positive functional gene group, whereas other GO classes close to the positive class in the GO hierarchy are expected to repeat the same signal at weaker signal levels [13,17]. Also, the true positive genes can come from one or a few independent (separate) functional groups. When we have signal from several independent gene groups, they can be considered to represent joint support against the null hypothesis, as they reduce the randomness of the gene list.

### Definition of the positive signal

In order to produce datasets with positive signal, we must first define the type of signal that we are embedding in the data. We are mainly interested in the surprising deviation from the expected number of class members in the reported gene list (for example, a cluster or a set of up-regulated genes), when comparing it to the reference group (usually the rest of the genome). Although there are various ways of measuring the deviation, hypergeometric distribution (HGD) based p-values have gained a lot of popularity. HGD methods measure the deviation, using the size of the cluster, class and the whole dataset (contingency table test conditional on the row and column sums). We have selected the reported p-value as a measure of the signal level that a user can feed into the data generation process.

There are currently three ways of calculating p-values from HGD: *a*) the distribution can be summed always towards the maximum value (the upper tail of the distribution, evaluation of over-representation [3]), *b*) the distribution can be summed by including all the outcomes with smaller probability (standard two-tailed test, often applied with Fisher's exact test), *c*) the distribution can be summed towards the closer tail (to minimum or maximum value) away from the mode of the distribution (one-tailed test) and multiplied with 2. Options *a *and *c *are demonstrated in the supplementary information in [5]. Here we have used the last option similarly to earlier work [5], with the exception that we multiply the obtained result by 2 (missing from the application in [5]) and set all the p-values larger than 1, after the multiplication, to be equal to 1. Still it should be noted that all the previous options (*a*, *b*, *c*) and also others, like the Log-likelihood-ratio based G-test, could be used during the signal definition.

### Definition of effective number and scaling for the multiple testing phenomena

Typical to any GO dataset is the large number of classes with complex correlation structure [12,13,25,26]. The large number of classes creates a multiple testing problem, in which we are more likely to see seemingly significant results arising from the random background distribution. Therefore it is standard, when testing multiple classes, to require a larger signal for significance. This affects similarly also the signal generation, as the signal has to be scaled accordingly to stand out from the background distribution. In order to compensate for this, the proposed method enhances the signal using the estimated number of independent classes (often referred as *effective number*, *N*_*eff*_) in the analyzed GO dataset (the actual details are shown later). This procedure is further modified when creating a signal with multiple signal classes.

A commonly used naïve estimate for *N*_*eff *_is to simply use the number of classes in the dataset. This often causes too strong a correction, as it does not take into consideration the correlations within the dataset [19]. An alternative is to estimate *N*_*eff *_using one of the many available methods [19,20,27]. Here we have used a simple estimate obtained by using the matrix rank. This standard linear algebra function gives the maximal number of linearly independent columns (i.e. GO classes) in the dataset.

Our current work selected three different simple methods for normalizing the signal with the number of independent signal classes and *N*_*eff*_. These were based on the False Discovery Rate (FDR [28]), Holm's method [29] and Bonferroni correction. Furthermore, one can omit the signal normalization totally and adjust the p-values before feeding them to the analysis.

Bonferroni correction is probably one of the most popular methods to address the multiple testing problem. It generates exactly the same correction independently from the number of the used signal classes, and therefore it behaves differently from the other methods, used here. It simply multiplies the reported p-value with the *N*_*eff*_: *p-value*_*bonf *_= p-value*N_*eff*_. The inverse function for this correction is simply:

(1)

where the *p-value *given by the user is simply divided with the *N*_*eff*_. FDR is another popular method that adjusts the p-values according to the risk of false discovery and its correction is based on the equation:

(2)

where *p-value*_*n *_stands for the *n*^*th *^p-value in the ordered ascending list of p-values and *n *is the rank. The minimum value of equation (2) over the ordered list of p-values is the q-value, proposed in [28]). It is interesting to note that for the smallest p-value, this equation turns out to be identical to Bonferroni's correction for multiple testing, *p-value*_*fdr *_= p-value*N_*eff*_. The inverse function of FDR correction is simply:

(3)

So this signal correction simply multiplies the user-selected p-value with the number of signal classes *n *divided by *N*_*eff*_. The obtained datasets, with the same user defined p-value and varying number of signal classes, should have similar FDR scores.

Another popular method to correct p-values in multiple testing is Holm's method [29]. Here the Bonferroni correction is sequentially modified in the ranked list of p-values so that the *N*_*eff *_decreases by 1 after every processed p-value in the sorted list. This generates for each rank *n *the following correction:

(4)

From this one can easily generate the inverse function

(5)

It should be noted that also other more sophisticated methods exist, like "truncated product method" [30], beta uniform mixture [31] and others [32,33]. These could be also used for normalization in the data generation process. However, we selected the methods above due to their simplicity.

### Ensuring the independence of the positive signals

**Figure 5:**
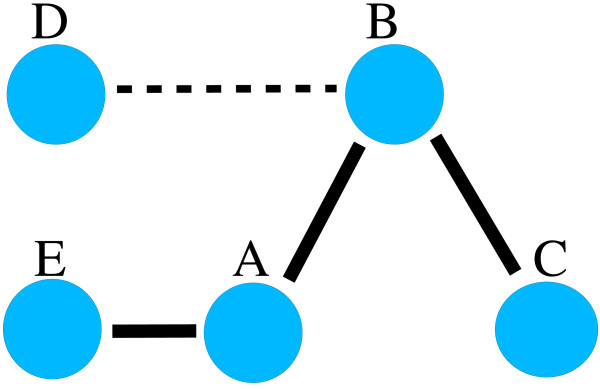
**A toy example of the filtering using correlation**. Circles represent various classes and thick line shows correlation between classes and dotted line an weak correlation. Classes have no correlation, when they lack a connecting line. Figure shows that if we have already class A among selected signal classes the first filtering discards classes B and E. Note that second filtering discards class C as we have a path of strong correlations through class B to it. Class D is left unfiltered. Positioning of circles in the figure is unrelated to the GO structure.

Due to the complex correlation structure of GO data sets, it is important to confirm that the selected positive classes are not referring to practically the same gene sets (dependent classes). We make this confirmation by looking at the pair-wise correlations between all GO categories. Note that this also has been used earlier for similar purpose [5] to filter correlating GO classes. Correlations larger than 0 and smaller than -0.2 are used here to exclude classes from the list of potential signal classes. Therefore this filtering allows only classes with weak negative correlation. We used correlation to filter out all the classes that show monitored correlation with any of the already selected potential signal classes (See Results for details). This step is demonstrated by filtering of classes A and C in the toy example shown by figure 5.

The complex hierarchical structure of GO places another requirement when ensuring the n copies of independent signals. Instead of multiple independent signals in the GO structure, the union of independent signal classes can actually match a class higher in the hierarchy, and therefore create a single stronger signal instead of two separate ones. To lessen this phenomenon a second level of filtering was included. At this level we exclude any class that can be connected, with mild correlations, to an already selected signal class via a third class. This is demonstrated by filtering of class E in the figure 3. These classes that do not need to have a strong direct pair-wise interaction with signal classes. A correlation stronger than 0.4 was used as a threshold here.

These filtering steps could also be based on some derivative of the graph distance between the classes in the GO graph. A drawback of such methods is that the 'distance' between two consecutive classes is not in reality a standard [25]. Also the graph distance does not show the correlations existing between the classes in totally different GO sub-graphs [25,26] The similarity or distance can be considered to change even when the analyzed total pool of genes is changed, say from total genome to genes found on an microarray. Therefore we consider the binary correlation of the GO classes (within the used GO dataset) to be the most reliable measure for the similarity of the signal within the two classes. Independence is ensured by omitting the classes having too strong correlation with already selected signal classes, during the selection of the new signal classes.

### Comparison of different GO analysis methods

We tested two types of enrichment analysis tools in our comparison. First, we compared two different tools which analyze the gene set in order to report a sorted list of enriched Gene Ontology terms [1] for the user. The first of these tools, DAVID [21], uses the standard approach where the user gets a list of GO terms sorted according to Fisher's Exact test p-value. DAVID uses GO database version Jan 2008. The second tool, Ontologizer [17], contains several algorithms for finding informative GO terms from the GO tree structure. For our comparison, we tested the parent-child [17] and topology-elimination [13] algorithms. As data, we used GO database version May 2009, and ENSEMBL gene GO annotations from ENSEMBL version 54.

Next, we compared methods that reduce the amount of information by clustering the list of genes into groups homogeneous in GO terms or by clustering the GO terms sharing similarity in the user given gene set. The first of these methods, GENERATOR [12], partitions genes into different numbers of clusters. We used an improved version of GENERATOR (Kurki et al., manuscript in submission) where different solutions are evaluated using the Akaike Information Criterion [34], and one of the solutions is chosen as representative. For each cluster, GENERATOR shows the GO terms that are over-represented in the cluster versus genome. GO terms that have enrichment *p *> 0.005 in the whole gene list are filtered out in order not to show GO terms not associated with the analyzed dataset to the user (see explanation from [12]). Clusters are sorted according to the lowest p-value in each cluster. As data, this tool uses GO database version from March 2009, and ENSEMBL gene GO annotations from ENSEMBL version 54. The other method, DAVID [18], combines GO terms based on their similarity using clustering. For each cluster of combined GO terms, a joint enrichment score is calculated, and the clusters are reported to the user as a sorted list according to this score. The versions of the annotations are the same as reported above. With all of the methods we used GO ontologies biological process and molecular function as data, as these were available in GENERATOR.

Throughout our analysis the compared programs may use different, older versions of GO and this might partially affect the results. However, the GO classes that were missing from the oldest GO version, used in our comparison, constituted only 0.43% of all the GO classes in our test dataset. Therefore, the different GO versions have most likely negligible effect on the results shown.

## Authors' contributions

PT and LH jointly developed the method. PP and PT did the evaluation of GO tools and all contributed to writing manuscript.

## Supplementary Material

Additional file 1 **Supplementary text**. We show the detailed descriptions of the developed functions. Click here for file
