# Supplementary material for: Generation of Gene Ontology benchmark datasets with various types of positive signal
Source: BMC Bioinformatics. 2009 Oct 7;10:319. doi: 10.1186/1471-2105-10-319 (PMC2762998; doi:10.1186/1471-2105-10-319)
Supplement: Additional file 1 — Supplementary text. We show the detailed descriptions of the developed functions. [file 1471-2105-10-319-S1.PDF]

1 Short Description

This is a supplementary for the BMC Bioinformatics publication 'Generation of Gene Ontology benchmark datasets with various types of positive signal'. It represents the workflow of POSGODA to aid the implementation of the method.

2 Work Flow of POSGODA

Here we represent the workflow for the positive data generation, in order to ease the implementation in different programming languages and with different settings.

Sketch of POSGODA work flow:

Input: Data dependent matrices and variables

- Binary GO N*M matrix (N genes in rows, M GO classes in columns) with 1 when the gene is a member of the class in question.
- M*M Binary correlation matrix, representing the GO class correlations
- Effective number N_{eff} that represents the estimated number of independent variables

---

User parameters

- Method of signal normalization for multiple signal classes
- Number of signal classes X
- Signal level for embedded p-value signal (with minimum and maximum values)
- Size of the generated positive data gene list

Output:

- Binary vector for N genes in the GO matrix with 1 for genes that belong to the gene list and 0 for genes that belong to the background set
- Column numbers for GO classes (in the original GO matrix) that represent the embedded positive signal

---

Work Flow for the selection of the signal classes

---

1. Adjust the p-value signal levels using the selected signal normalization, N$_eff$ and X
2. **Step A**: Create a random order for GO classes represented by the columns in the GO matrix
3. Run SelectIndependentGOClasses. Use the selected independent classes in the following steps.
4. **IF** the length of the list of obtained independent GO classes < 2*X
   (a) **GOTO** step D
5. **Step B**: Take the next GO class from the list of independent GO classes
6. **Step C**: Select a random signal that is between the adjusted minimum and maximum signals
7. Run FindClosestOverRepresentation. This looks for the over-representation that is closest to the given signal

8. **IF** the signal from the previous steps is between the minimum and maximum signal
    (a) Add the class to the SignalClassList
    (b) Add the obtained class outcome to the ClassOutcomeList
    (c) **IF** the required number of signal classes is obtained
        i. Program ends
    (d) **ELSE**
        i. **GOTO** step B
9. **ELSE**
    (a) **IF** this step has been visited 5 times
        i. **Step D**: **IF** this step has been visited already 20 times
            A. Program ends with an error
        ii. **ELSE**
            A. **GOTO** step A
    (b) **ELSE**
        i. **GOTO** step C

Output from the previous steps (when successful) for the following processes:

- SignalClassList
- ClassOutcomeList

Work Flow defining the artificial dataset

1. Define ResultBinaryVector as a vector of zeros of length N
2. **FOR** each signal class (i) in the SignalClassList **DO**
   (a) Create a random order for the genes in the GO matrix
   (b) Select genes that are members of class i for the following steps
   (c) Order the genes in the random list using the sum from the binary matrix over the columns of the other selected signal classes (*Using columns (1:i-1 i+1:X). First genes are not members of other signal classes, last genes are members of many other signal classes*)
   (d) Take the required number (Stated in ClassOutcomeList) of top sorted genes, and define them as 1 in the ResultBinaryVector
3. **IF** the sum(ResultBinaryVector) is less than the required size of the cluster
   (a) Create a list of genes that do not belong to any of the classes in the SignalClassList
   (b) Select randomly genes from the previous list to obtain the required cluster size
   (c) Define the selected genes as 1 in the ResultBinaryVector

Output from the whole POSGODA function

- ResultBinaryVector (representing the binary classification genes to sample list and background)
- SignalClassList (representing the GO classes that have the embedded signal)

The Previous work flow for POSGODA is represented in two parts. First part represents the subset where the independent signal classes are selected. Also the number of class members, required for the signal level, is defined here. The second part defines how the genes from signal classes are selected for the gene list. The first part is represented with several GOTO steps to simplify the work flow. Note that the first flow part ends at step 8.c.1. or with error at 9.a.1.1.

The following two flow charts represent the two sub functions used by the previous POSGODA work flow.

Flow Chart for SelectIndependentGOClasses

Input

- BinMatr = Binary Correlation M*M matrix for GO classes

Output

- SelectedIndependentClasses = list columns with independent signals

---

1. Define SelectedIndependentClasses = 1 (first class is taken automatically to signal classes)
2. Define IntermediateClasses as classes that have stronger correlation than 0.4 with any of the classes in the SelectedIndependentClasses
3. **FOR** each row i in BinMatr **DO**
   (a) Define min = minimum(BinMatr(i, SelectedIndependentClasses))
   (b) Define max = maximum(BinMatr(i, SelectedIndependentClasses))
   (c) Define max2 = maximum(BinMatr(i, IntermediateClasses))
   (d) **IF** min > 0.2 and max <= 0 and max2 < 0.4
      i. Add the row i to SelectedIndependentClasses
      ii. Add the classes that have stronger correlation than 0.4 with class i to the IntermediateClasses
4. **RETURN** SelectedIndependentClasses as a result

Flow Chart for FindClosestOverRepresentation

Input

- SignalLevel level of signal (-log p-value) that is targeted
- Size of the cluster
- Size of the class
- Size of the dataset

Output

- ClassMembersInCluster Number of class members in the cluster that gives the result closest to target signal level

1. Calculate the required Chi^2 score for the SignalLevel from Inverse of Chi^2 CDF
2. Calculate ClassMembersInCluster required to get the previous Chi^2 score
3. Define PreviousError = 1000 (*An arbitrary large value to force the following While to be true on the first time*)
4. Define X = Fisher exact test log p-value obtained with ClassMembersInCluster and size of the cluster, class and dataset.
5. Define CurrentError = absolute(X - SignalLevel)
6. **WHILE** CurrentError < PreviousError
   (a) Result = ClassMembersInCluster
   (b) **IF** X >= SignalLevel
      i. ClassMembersInCluster = ClassMembersInCluster + 1
   (c) **ELSE**
      i. ClassMembersInCluster = ClassMembersInCluster - 1
   (d) Define X = Fisher exact test log p-value obtained with ClassMembersInCluster and size of the cluster, class and dataset.
   (e) Define PreviousError = CurrentError
   (f) Define CurrentError = absolute(X - SignalLevel)
7. **RETURN** ClassMembersInCluster as a result
